# Legal obligation in the general population: face mask influence on endophthalmitis after intravitreal injection

**DOI:** 10.1007/s00417-022-05768-6

**Published:** 2022-08-06

**Authors:** Jonas Neubauer, Konstantinos Gklavas, Friederike Kortüm, Mariya Gosheva, Karl Ulrich Bartz-Schmidt, Focke Ziemssen

**Affiliations:** 1 Center for Ophthalmology, University Hospital Tuebingen, Elfriede-Aulhorn-Str. 7, 72076 Tuebingen, Germany; 2 Center for Ophthalmology, University Hospital Leipzig, Leipzig, Germany

**Keywords:** Covid-19, Post-injection endophthalmitis, Endophthalmitis, Intravitreal injection, Bacteria, Face mask

## Abstract

**Purpose:**

To investigate whether compulsory face masking in public life changes the incidence or pattern of post-injection endophthalmitis (PIE).

**Patients and methods:**

All injections of bevacizumab, ranibizumab, aflibercept, dexamethasone or triamcinolone between 01/01/2015 and 12/31/2021 at the University Eye Clinic of Tuebingen were included in this retrospective analysis. The injection procedure itself was unchanged since 2015 and included the use of a sterile drape covering the head up to the shoulders which prevents airflow toward the eye. Furthermore, all staff wore a face mask and gloves at all times. The two study periods were defined by the introduction of a compulsory face masking rule in public life (01/01/2015 until 04/27/2020 vs. 04/28/2020 until 12/31/2021).

**Results:**

A total of 83,543 injections were performed in the tertiary eye clinic, associated with a total of 20 PIE (0.024%, 1/4177 injections). Of these, thirteen PIE were documented during the pre-pandemic period (0.021%, 1/4773 injections) and seven PIE during the pandemic period (0.033%, 1/3071 injections). No significant difference in PIE risk was observed (*p* = 0.49), and there was no case of oral flora associated PIE.

**Conclusion:**

Although some potential confounders (wearing time, skin flora) could not be considered, there was no clear signal that the introduction of compulsory face masking in public life did alter the risk for PIE in our patient population. Three and six months after PIE, no difference in visual acuity was detectable between the two study periods.


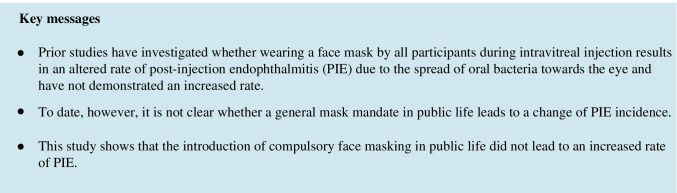


## Introduction

Endophthalmitis (EO) is one of the most serious complications of intravitreal injections. The most commonly found bacteria causing post-injection endophthalmitis (PIE) are coagulase-negative staphylococci, which are commonly found on human skin and conjunctiva [1]. It is known that endophthalmitis linked to oral flora is associated with a worse outcome compared to EO caused by coagulase-negative staphylococci. Therefore, precautions to prevent transmission of oral bacteria have been recommended [2–5].

The Covid-19 pandemic not only changed hygiene awareness and increased fear of viral infection, but was also accompanied by the need of a universal face mask use in most countries around the world [6]. Although it has been known for some time that the spread of aerosols can be reduced by the use of face masks, there are mixed data regarding the impact of bacterial spread in the ophthalmic setting [7, 8]. On the one hand, patients who do not wear a mask or who wear a loose mask have been shown to have an increased bacterial spread toward the eyes [9, 10]. On the other hand, Angaramo et al. have reported that wearing a loose mask does not increase the risk of bacterial colonization of the eye [11]. The hypothesis was that contaminated masks could affect the flora in the periocular region and/or the altered airflow could be associated with increased bacterial exposure in the sterile field. The aim of this study was therefore to investigate whether prolonged face mask wearing in everyday life, and not only during the injections resulted in an altered PIE risk.

## Methods

All intravitreal injections during the period 01/01/2015 to 12/31/2021 of bevacizumab, ranibizumab, aflibercept, dexamethasone and triamcinolone were included. Only EO cases caused by or related to prior intravitreal injections were included in the analysis. Cases with other causes of EO were excluded (i.e. postsurgical, bleb associated, endogenous, traumatic).

Since 2015, all staff, and from April 2020, all patients wore a face mask during the injection procedure. Nurses always wore gloves, and the physician who performed the injection wore sterile gloves. Intravitreal injections were performed following a standardized protocol [12]. Five drops of Oxybuprocain (4 mg/ml) were used for local anesthesia. The eye and the periocular area were disinfected with a povidone iodine solution (10% and 5%, for skin and conjunctiva respectively). After covering the eye with a sterile drape—which prevented any airflow from the mouth to the periocular area—a sterile lid speculum was inserted, and the drug was injected into the eye 3.5 mm from the limbus. After the orientation test of visual function (exclusion of acute loss of vision), a drop of lubricants was applied, and the speculum and sterile drape were removed. Neither topical antibiotics nor temporary bandage was used after the injection.

In case of suspected PIE, patients were treated with Gentamicin (5 mg/ml) and Moxifloxacin (5 mg/ml) eye drops every hour. All patients included in the study underwent a vitrectomy with vitreous biopsy and intravitreal injection of antibiotics (1 mg Ceftazidime and 1 mg Vancomycin). After surgery, intravenous treatment adapted to renal function with Ceftazidime, Imipenem and Cilastin was initiated and continued for at least 7 days. A culture positive PIE was defined by growth of bacteria in the bacterial culture. Some of the negative samples were re-examined by PCR. If a specific bacterial strain was detected, the sample was also classified as culture positive PIE, although no antibiogram could be obtained.

Visual acuity was analyzed using LogMAR. Low visual acuity values like counting fingers, hand movement and light perception were converted to LogMAR 2.0, 2.3 and 2.7, respectively. Statistical analysis was performed using R and Prism 6, GraphPad. Fisher’s test or Chi-square test with Yates’ correction and the Mann–Whitney *U*-test were used, and statistical significance was assumed for *p* < 0.05.

This study was performed in line with the principles of the Declaration of Helsinki. Approval was granted by the Ethics Committee of the University of Tuebingen (Project Number 145/2022BO2).

## Results

During the study period, 83,543 injections were performed in the tertiary center, associated with a total of 20 PIE (0.024%, 1/4,77 injections) (Table 1). Of these, 62,044 injections were performed in the period between 01/01/2015 and 04/27/2020 and 21,499 in the period between 04/28/2020 and 12/31/2021, with 13 PIE (0.021%, 1/4773 injections) and 7 PIE (0.033%, 1/3071 injections), respectively. No significant difference in PIE risk was observed between the time periods (*p* = 0.49). During the pre-pandemic period, 30,053 Bevacizumab, 16,998 Aflibercept, 13,156 Ranibizumab, 855 Dexamethasone and 982 Triamcinolone injections were administered while during the pandemic period, 12,203 Bevacizumab, 5523 Aflibercept, 3152 Ranibizumab, 395 Dexamethasone and 226 Triamcinolone injections were carried out.

**Table 1:** Summary of documented intravitreal injections and registered post-injection-endophthalmitides (PIE)

	Pre-pandemic	Pandemic	*p* value
Intravitreal injection			
Bevacizumab	4 (31%)	4 (57%)	0.24
Aflibercept	3 (23%)	3 (43%)	0.16
Ranibizumab	3 (23%)	-	-
Dexamethasone	-	-	-
Triamcinolone	3 (23%)	-	-
Total injections	62,044	21,499	
PIE	13	7	0.49

The median age of patients with PIE was 76 years (IQR 72.25–81.75). In the pre-pandemic group, the median age was 76 years (IQR 72–79.5), and in the pandemic group it was 80 years (IQR 72–82) (*p* = 0.45). On median, patients had previously received 29 injections (IQR 5.5–47.5) in the pre-COVID group and 20 injections (IQR 12–28) in the COVID group (*p* = 0.83). In the pre-pandemic group, PIE was associated with neovascular AMD in 46% (*n* = 6), veno-occlusive disease in 46% (*n* = 6) and diabetic macular edema in 8% (*n* = 1). In contrast, in the pandemic group, 86% (*n* = 6) of PIE occurred in patients with neovascular AMD and 14% (*n* = 1) in diabetic macular edema.

The time between injection and admission to the emergency clinic demonstrated no significant difference between the two groups (pre-pandemic: median 4 days (IQR 3.5–8.5) and pandemic: median 9 days (IQR 3–15) (*p* = 0.71)).

VA on the day of intravitreal injection was median LogMAR 0.3 (IQR 0.15–1.25) in the pre-pandemic group and median LogMAR 0.4 (IQR 0.3–0.7) in the pandemic group (*p* = 0.57). On the day of emergency presentation, the pre-COVID group had a median visual acuity of LogMAR 2.3 (IQR 1.75–2.3), and the COVID group had a median visual acuity of LogMAR 2.3 (IQR 1.7–2.3) (*p* = 0.96). There was also no significant difference at 3 and 6 months post-injection (*p* = 0.28 and *p* = 0.45, respectively).

Of the twenty vitreous samples examined, a positive bacterial culture was confirmed in fourteen cases, and an antibiogram was obtained in twelve cases. A summary of these results can be found in Table 2.

**Table 2:** Summary of all PIE vitreous samples

	Patient ID	Causative injection	Pathogen	Gentamicin	Oxacillin	Cefazolin	Tetracyclin	Cotrimoxazole	Erythromycin	Clindamycin	Ciprofloxacin	Vancomycin	Rifampicin
Pre-pandemic	1	Ranibizumab	S. epidermidis	R	R	R	I	S	R	S	R	S	S
	2	Triamcinolone	S. epidermidis	S	S	S	S	S	S	S	R	S	S
	3	Bevacizumab	S. epidermidis	S	S	S	I	I	R	R	R	S	S
	4	Ranibizumab	S. epidermidis	S	S	S	I	S	S	S	S	S	S
	5	Triamcinolone	S. epidermidis	S	S	S	S	R	S	S	S	S	S
	6	Aflibercept	S. epidermidis	S	S	S	I	S	R	S	S	S	S
	7	Bevacizumab	S. aureus	S	S	S	S	S	S	S	I	S	S
	8 + 9	Ranibizumab, Aflibercept	S. epidermidis *	N/A									
	10–13	Bevacizumab, Aflibercept, Bevacizumab, Triamcinolone	Unknown										
Pandemic	14	Aflibercept	S. epidermidis	S	R	R	S	S	S	S	R	S	S
	15	Aflibercept	S. epidermidis	S	N/A	R	R	S	R	N/A	I	S	S
	16	Aflibercept	S. lugdunensis	R	S	S	S	S	S	S	S	S	S
	17	Bevacizumab	S. aureus	S	S	S	S	S	S	S	S	S	S
	18	Bevacizumab	S. aureus	S	S	S	S	S	S	S	I	S	S
	19 + 20	Bevacizumab, Bevacizumab	Unknown	N/A									

*S* sensible, *I* inhibition, *R* resistant, *S.* Staphylococcus, *N/A* not available

^*^Detection of the bacterial strain via PCR

## Discussion

This study investigates whether the introduction of universal compulsory face masking in public life leads to increased rates of PIE. During the Covid-19 pandemic, various measures were taken to contain the spread of the virus, with mandatory face masking introduced in most countries. In Germany, this was introduced on the 27^th^ of April 2020 and included the wearing of a mouth-nose mask in all public buildings, businesses and public transportation [13]. Due to its far-reaching impact on people’s daily lives, it is of great interest to determine whether the introduction of a general face mask mandate leads to an increased risk of PIE. This study examined 83,543 injections, with 62,044 administered before and 21,499 during the pandemic, while a face mask law was in place.

During the Covid-19 pandemic, a delayed emergency department presentation was reported for medical emergencies, including ophthalmology [14–16]. In contrast to these findings, we did not observe a delayed presentation of PIE patients during the pandemic which likely also contributed to the fact that visual acuity after 3 and 6 months was comparable between the two groups.

Previous research has focused on the incidence of PIE following the introduction of face masking by both patients and physicians during the intravitreal injection itself, rather than the influence of face masking in public life on PIE.

It is known that patients wearing loose face masks during injections may have an increased dispersion of oral bacteria toward the periocular area [9, 10]. However, concerns that this may lead to increased rates of PIE or more frequent infections with oral flora associated EO have not been confirmed [17, 18]. The time periods studied in both publications depended on the use of face masks during the injection procedure rather than the introduction of a general mask mandate in public life, which was not taken into consideration. Likewise, a recently published IRIS Registry study from the USA was unable to address this question, as the individual states had very diverse and variable legislation regarding compulsory face masking in public life [19]. In addition, the time periods examined during the pandemic were relatively short in all three previously mentioned studies, so that influences that may only have set in after longer mask wearing may not have been captured.

A number of factors play a role in influencing the risk of PIE, and these have been discussed before in detail [4, 20, 21]. Nevertheless, eyes with blepharitis are known to be at higher risk of PIE, and it is assumed that this is due—at least in part—to the increased and altered bacterial load on the conjunctiva [22]. Furthermore, it is recognized that wearing face masks increases the incidence of dry eye disease and that these patients have an altered conjunctival microbiome [23, 24]. In addition, wearing a loose mask can cause orally associated bacteria to enter the periocular area during breathing and may contribute to the disruption of the microbiome [9, 10]. Changes in the conjunctival microbiome make the eyes more susceptible to infection, and there is therefore reason to be concerned that prolonged daily wear of a face mask may reduce the eye’s natural defenses against pathogenic bacteria [25].

In contrast to the three studies described previously, Blom et al. have shown that the introduction of a general mask requirement could potentially lead to an increased risk of PIE [26]. However, it should be noted that the number of injections during the period of mandatory masking was only 14,649 in that study. Despite the fact that our study included many more injections and that face masks were worn longer and more intensively in Germany than in Norway, we were unable to demonstrate an increased risk of PIE in our study population [27, 28].

A main strength of our study is that there has been no relevant change in our preparation and injection protocol since 2015. Even before other guidelines such as the statement by the EURETINA expert panel recommended it, the nursing staff and physicians always wore a face mask and gloves [4]. Since a self-adhesive and sterile drape covering the entire head up to the shoulders has already been used since 2015, bacterial dispersion from the patient’s mouth to the periocular region during the injection procedure can be excluded. This is also supported by the fact that none of our PIE patients had bacteria associated with the oral flora.

There are several limitations to this study. Primarily, the rarity of PIE must certainly be mentioned here. Because such events can occur in clusters, large numbers and long observation periods are necessary to allow reasonably reliable conclusions. Despite the large patient population (83,543 injections), the sample may be too small to detect a small change in risk and exclude relevant confounders. Because of the rarity of PIE, only a limited number could be used as the basis for the descriptive statistics, in contrast to a prospective study with appropriate case number planning and formulated hypothesis. Other study limitations include unavailable information. Although the influence of wearing time and other hygienic characteristics on bacterial colonization of the periocular region is known, we were unable to analyze how many minutes per day and which type of mask (surgical or FFP2) was worn by patients because of the study’s retrospective nature [29–32]. It should also be noted that the results of this study may not be generalizable to other countries, as adherence to measures such as face masks during the pandemic differs between countries [27].

In summary, this study found no evidence of an increased risk of PIE from a universal mask mandate. Nevertheless, patients should continue to be educated about the importance of hygiene after intravitreal injections to minimize the risk of PIE. In addition, consistent use of artificial tears should be advised during prolonged face mask wear to counteract the development of dry eye disease.

## References

[1] Dossarps D, Bron AM, Koehrer P, Aho-Glele LS, Creuzot-Garcher C, net F (2015). Endophthalmitis after intravitreal injections: incidence, presentation, management, and visual outcome. Am J Ophthalmol.

[2] Doshi RR, Leng T, Fung AE (2012). Reducing oral flora contamination of intravitreal injections with face mask or silence. Retina.

[3] Garg SJ, Dollin M, Storey P, Pitcher JD, Fang-Yen NH, Vander J, Hsu J, Post-Injection Endophthalmitis Study T (2016). Microbial spectrum and outcomes of endophthalmitis after intravitreal injection versus pars plana vitrectomy. Retina.

[4] Grzybowski A, Told R, Sacu S, Bandello F, Moisseiev E, Loewenstein A, Schmidt-Erfurth U, Euretina B (2018). 2018 Update on intravitreal injections: Euretina Expert Consensus Recommendations. Ophthalmologica.

[5] Shimada H, Hattori T, Mori R, Nakashizuka H, Fujita K, Yuzawa M (2013). Minimizing the endophthalmitis rate following intravitreal injections using 0.25% povidone-iodine irrigation and surgical mask. Graefes Arch Clin Exp Ophthalmol.

[6] Kortuem FC, Ziemssen F, Kortuem KU, Kortuem C (2022). International survey on COVID-19 pandemic: personal protective measures during fundus examination. Acta Ophthalmol.

[7] Leung NHL, Chu DKW, Shiu EYC, Chan KH, McDevitt JJ, Hau BJP, Yen HL, Li Y, Ip DKM, Peiris JSM, Seto WH, Leung GM, Milton DK, Cowling BJ (2020). Respiratory virus shedding in exhaled breath and efficacy of face masks. Nat Med.

[8] Milton DK, Fabian MP, Cowling BJ, Grantham ML, McDevitt JJ (2013). Influenza virus aerosols in human exhaled breath: particle size, culturability, and effect of surgical masks. PLoS Pathog.

[9] Hadayer A, Zahavi A, Livny E, Gal-Or O, Gershoni A, Mimouni K, Ehrlich R (2020). Patients wearing face masks during intravitreal injections may be at a higher risk of endophthalmitis. Retina.

[10] Patel SN, Mahmoudzadeh R, Salabati M, Soares RR, Hinkle J, Hsu J, Garg SJ, Regillo CD, Ho AC, Cohen MN, Khan MA, Yonekawa Y, Chiang A, Gupta OP, Kuriyan AE (2021). Bacterial dispersion associated with various patient face mask designs during simulated intravitreal injections. Am J Ophthalmol.

[11] Angaramo S, Law JC, Maris AS, Schmitz JE, Liu Y, Chen Q, Chomsky A (2021). Potential impact of oral flora dispersal on patients wearing face masks when undergoing ophthalmologic procedures. BMJ Open Ophthalmol.

[12] Jaissle GB, Szurman P, Bartz-Schmidt KU, German Retina S, German Society of O, German Professional Association of O (2005). [Recommendation for the implementation of intravitreal injections—statement of the German Retina Society, the German Society of Ophthalmology (DOG) and the German Professional Association of Ophthalmologists (BVA)]. Klin Monbl Augenheilkd.

[13] Bundesregierung-Deutschland (2020) Gesetz zur Verhütung und Bekämpfung von Infektionskrankheiten beim Menschen (Infektionsschutzgesetz - IfSG) § 28a Besondere Schutzmaßnahmen zur Verhinderung der Verbreitung der Coronavirus-Krankheit-2019 (COVID-19). https://www.gesetze-im-internet.de/ifsg/__28a.html . Accessed 01.01.2022

[14] Au SCL, Ko CKL (2021). Delayed hospital presentation of acute central retinal artery occlusion during the COVID-19 crisis: the HORA study brief report No. 4. Indian J Ophthalmol.

[15] Awad M, Poostchi A, Orr G, Kumudhan D, Zaman A, Wilde C (2021). Delayed presentation and increased prevalence of proliferative vitreoretinopathy for primary rhegmatogenous retinal detachments presenting during the COVID-19 pandemic lockdown. Eye (Lond).

[16] Mafham MM, Spata E, Goldacre R, Gair D, Curnow P, Bray M, Hollings S, Roebuck C, Gale CP, Mamas MA, Deanfield JE, de Belder MA, Luescher TF, Denwood T, Landray MJ, Emberson JR, Collins R, Morris EJA, Casadei B, Baigent C (2020). COVID-19 pandemic and admission rates for and management of acute coronary syndromes in England. Lancet.

[17] Patel SN, Tang PH, Storey PP, Wolfe JD, Fein J, Shah SP, Chen E, Abbey A, Ferrone PJ, Shah CP, Liang MC, Stem MS, Ali Khan M, Yonekawa Y, Garg SJ, Writing committee for the Post-Injection Endophthalmitis Study G (2021). The influence of universal face mask use on endophthalmitis risk after intravitreal anti-vascular endothelial growth factor injections. Ophthalmology.

[18] Naguib MM, Ghauri S, Mukhopadhyay A, Schefler AC (2021). Endophthalmitis after intravitreal injections during the Covid-19 pandemic with implementation of universal masking. Retina.

[19] Lum F, Li S, Liu L, Li C, Parke DW, Williams GA (2022). The pandemic is not associated with endophthalmitis decrease after anti-vascular endothelial growth factor injections. Ophthalmology.

[20] Shah CP, Garg SJ, Vander JF, Brown GC, Kaiser RS, Haller JA, Post-Injection Endophthalmitis Study T (2011). Outcomes and risk factors associated with endophthalmitis after intravitreal injection of anti-vascular endothelial growth factor agents. Ophthalmology.

[21] Stem MS, Rao P, Lee IJ, Woodward MA, Faia LJ, Wolfe JD, Capone A, Covert D, Dass AB, Drenser KA, Garretson BR, Hassan TS, Margherio A, Oh KT, Raephaelian PV, Randhawa S, Sneed S, Trese MT, Yedavally S, Williams GA, Ruby AJ (2019). Predictors of endophthalmitis after intravitreal injection: a multivariable analysis based on injection protocol and povidone iodine strength. Ophthalmol Retina.

[22] Lyall DA, Tey A, Foot B, Roxburgh ST, Virdi M, Robertson C, MacEwen CJ (2012). Post-intravitreal anti-VEGF endophthalmitis in the United Kingdom: incidence, features, risk factors, and outcomes. Eye (Lond).

[23] Krolo I, Blazeka M, Merdzo I, Vrtar I, Sabol I, Petric-Vickovic I (2021). Mask-associated dry eye during COVID-19 pandemic—how face masks contribute to dry eye disease symptoms. Med Arch.

[24] Willis KA, Postnikoff CK, Freeman A, Rezonzew G, Nichols K, Gaggar A, Lal CV (2020). The closed eye harbours a unique microbiome in dry eye disease. Sci Rep.

[25] Petrillo F, Pignataro D, Lavano MA, Santella B, Folliero V, Zannella C, Astarita C, Gagliano C, Franci G, Avitabile T, Galdiero M (2020) Current evidence on the ocular surface microbiota and related diseases. Microorganisms 8(7). 10.3390/microorganisms8071033 10.3390/microorganisms8071033 PMC7409318 32668575

[26] Blom K, Bragadottir R, Sivertsen MS, Moe MC, Jorstad OK (2021). Mask use by patients in the context of COVID-19 can increase the risk of postinjection endophthalmitis. Acta Ophthalmol.

[27] Statista (2021) How often have you worn a face mask outside your home to protect yourself or others from coronavirus (COVID-19)? . https://www.statista.com/statistics/1114375/wearing-a-face-mask-outside-in-european-countries/. Accessed 01.01.2022

[28] Kortuem FC, Ziemssen F, Kortuem KU, Kortuem C (2021). The role and views of ophthalmologists during the COVID-19 pandemic. Clin Ophthalmol.

[29] Marin-Nieto J, Reino-Perez C, Santillana-Cernuda G, Diaz-Bernal JM, Luque-Aranda R, Garcia-Basterra I (2021). Face mask contamination during Covid-19 pandemia. A study on patients receiving intravitreal injections. Retina.

[30] Guellich A, Tella E, Ariane M, Grodner C, Nguyen-Chi HN, Mahe E (2021). The face mask-touching behavior during the COVID-19 pandemic: observational study of public transportation users in the greater Paris region: the French-mask-touch study. J Transp Health.

[31] Delanghe L, Cauwenberghs E, Spacova I, De Boeck I, Van Beeck W, Pepermans K, Claes I, Vandenheuvel D, Verhoeven V, Lebeer S (2021). Cotton and surgical face masks in community settings: bacterial contamination and face mask hygiene. Front Med (Lausanne).

[32] Allison ALA-DE, Bawn M, Casas Arredondo M, Chau C, Chandler K et al (2021) The impact and effectiveness of the general public wearing masks to reduce the spread of pandemics in the UK: a multidisciplinary comparison of single-use masks versus reusable face masks. UCL Open Environ 3(01). 10.14324/111.444/ucloe.000022 10.14324/111.444/ucloe.000022 PMC10208332 37228803

